# Cordycepin Inhibits Triple-Negative Breast Cancer Cell Migration and Invasion by Regulating EMT-TFs SLUG, TWIST1, SNAIL1, and ZEB1

**DOI:** 10.3389/fonc.2022.898583

**Published:** 2022-06-14

**Authors:** Chunli Wei, Md. Asaduzzaman Khan, Jiaman Du, Jingliang Cheng, Mousumi Tania, Elaine Lai-Han Leung, Junjiang Fu

**Affiliations:** ^1^ Key Laboratory of Epigenetics and Oncology, The Research Center for Preclinical Medicine, Southwest Medical University, Luzhou, China; ^2^ State Key Laboratory of Quality Research in Chinese Medicine/Macau Institute for Applied Research in Medicine and Health, Macau University of Science and Technology, Macao, Macao SAR, China

**Keywords:** cordycepin, triple-negative breast cancer (TNBC), Twist1, Slug, cell migration and invasion, cancer therapeutics

## Abstract

Cancer metastasis is the most important cause of cancer-related death, and epithelial-to-mesenchymal transition (EMT) plays crucial roles in cancer metastasis. Cordycepin (CD) is highly enriched in the medicinally used *Cordyceps* mushroom. In this study, we conducted the antimetastatic activities of CD, specifically focusing on its regulatory effects on EMT-inducing transcription factors (EMT-TFs) in triple-negative breast cancer (TNBC). Our study showed CD to inhibit the growth, migration, and invasion of BT549 and 4T1 cancer cell lines, by employing cell viability assay and real-time cell analyses. The protein levels of N-Cadherin and E-Cadherin, as well as their transcription factors TWIST1, SLUG, SNAIL1, and ZEB1 in BT549 and 4T1 cells, were estimated by Western blot assays. Results from dual-luciferase reporter assays demonstrated that CD is capable of inactivating the EMT signaling pathway by inhibiting TWIST1 and SLUG expression. Furthermore, *in vivo* studies with mice carrying cancer cell-derived allograft tumors showed the inhibitory effect of CD on cancer cell growth and metastasis. Furthermore, the additive/synergistic anti-metastasis effect of CD and thymoquinone (TQ), another natural product with promising anticancer roles, was demonstrated by combinational treatment. The results from this research indicate that CD would be a promising therapeutic molecule against TNBC by targeting EMT-TFs, possibly in SLUG, TWIST1, SNAIL1, and ZEB1.

## Introduction

*Cordyceps* mushrooms have long been used as Traditional Chinese Medicine (TCM). Among the 400 species of this genus, *C. sinensis* and *C. militaris* were reported as the most widely used medicinal products against multiple life-threatening diseases, including cancer (1–3). More attention has been drawn to the scientists in basic research in the past few decades, and the major functional anticancer component in both *C. sinensis* and *C. militaris* is known as ‘Cordycepin’ (CD) (3′-deoxyadenosine), which is a derivative of the adenosine nucleotide differing from adenosine due to the absence of oxygen at the 3′-position of its ribose moiety (4, 5). The underlying mechanisms of CD’s anticancer/antimetastatic activities include targeting various intracellular targets of nucleic acids (DNA/RNA) and proteins involved in cellular apoptosis, cell cycle, and other signaling pathways (2, 6–8). The latest research also found the therapeutic potential of CD against COVID-19 virus or viral receptor as a novel therapeutic strategy (9).

Triple-negative breast cancer (TNBC) is diagnosed based on the negativity of all three receptors that are found in other breast cancer types: estrogen receptor (ER), progesterone receptor (PR), and human epidermal growth factor (HER-2). Although TNBC only represents 15%–20% of all breast cancer types, there are limited options for the treatment of this kind of cancer because these cells are responsive to neither hormone nor targeted therapy (10). Therefore, it is urgent to develop effective therapeutic strategies targeting TNBC.

Epithelial-to-mesenchymal transition (EMT) plays a crucial role in cancer metastasis. The up-regulated expression of N-Cadherin and/or down-regulated expression of E-Cadherin are common features of enhanced EMT and metastasis. In addition, a panel of transcription factors (TFs) including TWIST1, SLUG, SNAIL1, and ZEB1 is known to be involved in regulating cadherin expressions and EMT (11–13). Therefore, targeting either cadherin directly or the involved TFs indirectly are potential strategies to stop or slow down EMT and metastasis (14).

In this study, we explored the anticancer and antimetastatic activities of CD, specifically focusing on its regulatory effects on EMT-inducing transcription factors (EMT-TFs). The roles and mechanisms for combination treatment by CD and another promising natural product thymoquinone (TQ) in TNBC cells are also explored.

## Materials and Methods

### Reagents and Cells

CD was purchased from Chengdu Must Bio-Technology Co., Ltd. (China). Dimethyl sulfoxide (DMSO) was purchased from Sigma (St. Louis, MO, USA). MTT reagent was purchased from Acros Organics (Fair Lawn, NJ, USA). TWIST1 (ab175430) antibody was purchased from Abcam (Cambridge, MA, USA). SLUG (SC-166476), ZEB1 (SC-25388), and SNAI1 (SC-10432) antibodies were purchased from Santa Cruz (Dallas, TX, USA). N-Cadherin (13A9) and E-cadherin (24E10) antibodies were purchased from Cell Signaling (Danvers, MA, USA). Notch1 antibody (14-5785) was purchased from Affymetrix (Santa Clara, CA, USA). Tubulin antibody, β-actin antibody, anti-rabbit secondary antibodies, and anti-mouse secondary antibodies were purchased from Sigma (USA). The luciferase assay promoter for TWIST1 was reported previously (15). The luciferase assay promoter for SLUG and Slug_pGL2 (#31695) was obtained from Addgene (Watertown, MA, USA) (https://www.addgene.org/31695/). Nuclear and Cytoplasmic Protein Extraction Kit was purchased from Beyotime (P0027, Beijing, China).

DMEM, RPMI 1640 medium, fetal bovine serum (FBS), and trypsin were bought from Gibco (USA). Human breast cancer cell line BT549 was maintained at 37°C with 5% CO_2_ in 1640 media. Mouse breast cancer cell line 4T1, 168FARN, and human cervical cell line HeLa were maintained at 37°C with 5% CO_2_ in DMEM media. BT549, 4T1, and HeLa cell lines were obtained from the American Type Culture Collection (ATCC, USA).

### Cell Viability Assay

3-(4,5-Dimethylthiazol-2-yl)-2,5-diphenyltetrazolium bromide (MTT) assay was used to examine the cell viability (15, 16). Cells were plated with 3,000~5,000 cells/well in a 96-well microplate for overnight. The cells were treated with various concentrations of CD for 24, 48, and 72 h, respectively. Then, in each well, 10 µl of MTT reagent was added and incubated for 4 h at 37°C. After removing the medium and adding 100 μl DMSO with shaking for 10 min, the absorbance at 570 and 650 nm was recorded from the microplate reader (Thermo Scientific, Waltham, MA, USA). Each experiment was repeated for three times.

### Cell Growth, Migration, and Invasion Assay

The cell growth, migration, and invasion were examined by the real-time cell analyzer (xCELLigence RTCA DP, Roche, Germany) (17, 18). For cell growth, cell suspensions with 100 μl (2 × 10^4^ cells/ml) media were seeded on each of the 16-well E-plate. For cell migration and invasion, the lower chamber wells of CMI plates were filled with 10% serum supplemented media, and 100 µl of cell suspensions (2 × 10^4^ cells/ml) in medium without FBS was added into the upper wells. Matrigel was seeded on the upper chamber wells of CMI plates before invasion assay. Five micromoles or 10 μM of CD or DMSO was added after 10 h (cell growth) or 20 h (cell migration and invasion) of cell growth (16).

### Western Blotting

Cells with CD treatment were lysed by 1× RIPA buffer and 1 mM phenylmethylsulfonyl fluoride (PMSF). Cell lysates (proteins in solution) were separated on 10% SDS-PAGE with 1× running buffer at 100 V for the complete protein separation. After transferring to the PVDF membrane, the membrane was blocked with 5% non-fat milk for 2~3 h at room temperature. Then, the primary antibodies were incubated at 4°C overnight. Next day, after being washed with TBST for three times and incubated in secondary antibodies for 1~2 h at room temperature, the membranes were detected after washing with TBST for the intensity of each band by an imaging scanner (16). Each experiment was repeated for three times.

### Luciferase Reporter Assay

Luciferase reporter assay was performed by using TWIST1 and SLUG promoter reporter genes. BT549 cells were transfected with the TWIST1 or SLUG promoter reporter. 5, 15 and 45 μM of CD or DMSO were added after 24 h of transfection. Luciferase activity was measured with the Dual-Glo assay (Promega, USA). The data are presented as the mean ± SD from at least three samples per data point.

### RNA Extraction and Quantitative RT-PCR

4T1 or BT549 cells in a six-well plate were treated with different concentrations (0, 5, 10, and 20 μM) of CD for 24 h. Total RNA was extracted with mRNA extraction kit (TRANGEN, Beijing, China) and reverse-transcribed into cDNA. Semiquantitative RT-PCR for the *TWIST1* gene was performed using primers (forward primer: 5′-gtccgcagtcttacgaggag-3′, reverse primer: 5′-cacgccctgtttctttgaat-3′), that for the *SLUG* gene was performed using primers (forward primer: 5′-catgccattgaagctgaaaa-3′, reverse primer: 5′-gcagtgagggcaagaaaaag-3), that for the *SNAIL1* gene was performed using primers (forward primer: 5′-aaggaccccacatccttctc-3′, reverse primer: 5′-ggagcttcccagtgagtctg-3′), and that for the *ZEB1* gene was performed using primers (forward primer: 5′-ggagccttgatgtggtagga-3′, reverse primer: 5′-gcttgactttcagccctgtc-3′). GAPDH was served as internal control. Quantitative RT-PCR was also applied for *TWIST1*, *SLUG*, *SNAIL1*, and *ZEB1*. Each experiment was repeated for three times.

### Mouse Allograft Assays and Histology

Studies for the animal model were in strict compliance with institutional animal care guidelines and conducted according to the protocols of the Southwest Medical University board. 4T1 mouse breast cancer cells were injected into the mammary fat pad of female BALB/c mice to establish breast cancer models (17). Three days later after injection, the mice were randomly divided into three groups for treatment with CD (0, 25 mg/kg, 75 mg/kg). Tumor size was measured every 5 days, and the mice were sacrificed at the end of the 30-day treatment to detect the effect of CD on breast tumor metastasis *in vivo.* Tumor tissues were fixed in 10% formalin for 24 h, embedded in paraffin, and sliced every 5 µm, after dewaxing in xylene, dehydrating in decreasing concentrations of alcohol, and staining with hematoxylin and eosin.

### Statistical Analysis

All data were analyzed by one-way ANOVA using SPSS 20 and GraphPad Prism 9. p values < 0.05 were considered significantly different. *p < 0.05, **p < 0.01.

## Results

### Cordycepin Inhibits Growth, Migration, and Invasion of TNBC Cells

**Figure 1:**
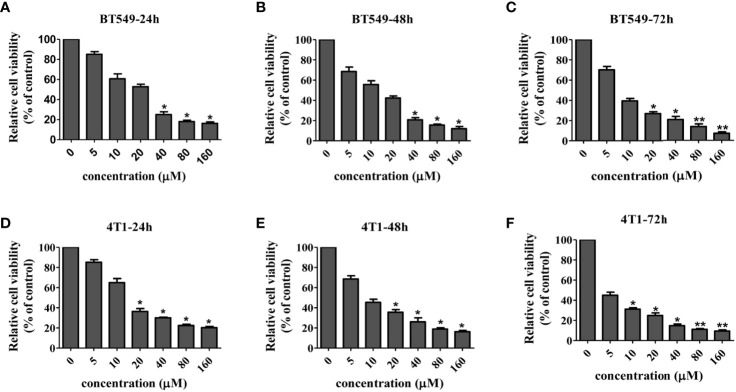
Cellular toxicity of cordycepin. Cancer cells were treated with different concentrations of CD for 24/48/72h, and MTT assay was performed. BT549 cells were treated with different concentrations of CD for 24h/48h/72h **(A–C)**. 4T1 cells were treated with different concentrations of CD for 24h/48/72h **(D–F)**. Results are presented as mean±SD, which shows that CD dose dependently decreases the cell viability. *P<0.05, **P<0.01 (N=3).

**Figure 2:**
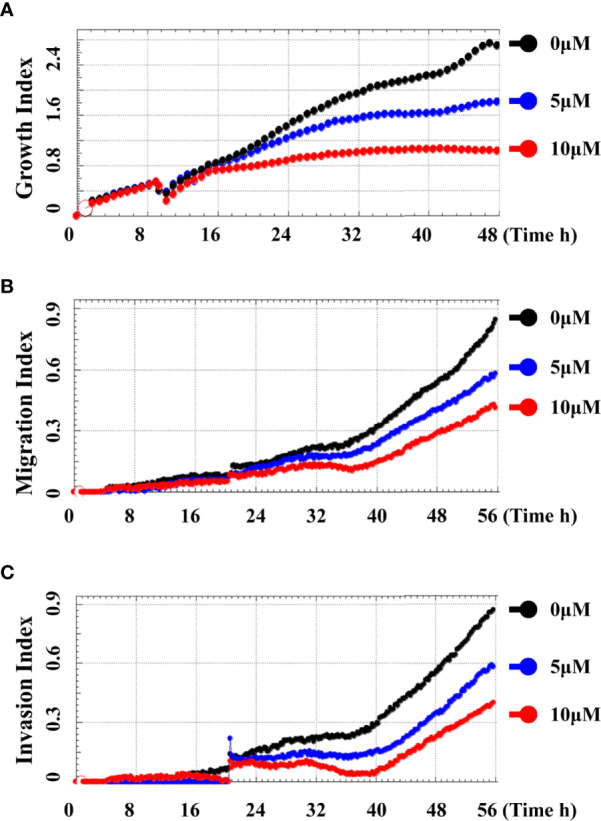
Cordycepin inhibits cell growth, migration, and invasion in TNBC. Cells were analyzed by real time cell analyzer after treating with different concentrations of CD. CD dose dependently inhibited the growth **(A)**, migration **(B)**, and invasive **(C)** in 4T1 cells.

In order to systematically determine the specific effects of CD on the TNBC cells, we first determined the cytotoxicity of CD in human (BT549) and mouse (4T1) triple-negative breast cell lines by MTT assays. **(Figures 1A–F)** and **Table 1** show that CD can inhibit both BT549 and 4T1 cell growth in a dose-dependent fashion. The cytotoxic effect of CD was the same as on HeLa cells (**Supplementary Figure 1**). Next, we conducted a systemic estimation of the effect on cancer cell migration and invasion in 4T1 cells. **Figure 2** shows that CD is capable of inhibiting growth, migration, and invasion of 4T1 cells (**Figures 2A–C**). In addition, the inhibitory effects of CD on cell growth, migration, and invasion were also confirmed in HeLa cells (**Supplementary Figure 2**), suggesting that the above observed anticancer effects of CD are likely not limited to the TNBC, rather it might be effective against different cancers.

**Table 1:** The IC_50_ values of cordycepin in breast cancer cell lines.

Cell types	IC_50_ values at 24 h (μM)	IC_50_ values at 48 h (μM)	IC_50_ values at 72 h (μM)
BT549	19.12 ± 3.315	12.54 ± 1.88	8.83 ± 1.695
4T1	18.38 ± 2.165	10.46 ± 1.935	3.43 ± 0.957

### Cordycepin Regulates EMT-Associated Protein Expression

**Figure 3:**
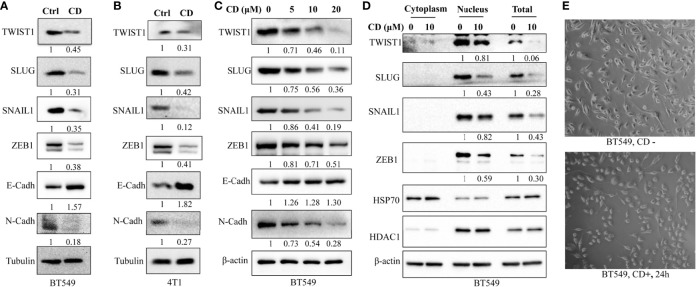
Effects of cordycepin on the expression of EMT-associated proteins. **(A)** EMT-TF expression in BT549. **(B)** EMT-TF expression in 4T1. For protein level expression analysis, cancer cells were treated with 10 µM of CD for 24 h, cellular protein was lysated, and Western blot analysis was performed. Results are repeated twice. **(C)** EMT-TF expression in BT549 treated with the indicated concentration of CD. For protein level expression analysis, cancer cells were treated with 0, 5, 10, and 20 µM of CD for 24 h, cellular protein was lysated, and Western blot analysis was performed. Results are repeated twice. **(D)** CD reduces EMT-TF nuclear localizations. BT549 treated without or with the indicated concentration of CD, proteins from cytoplasm, nucleus, or total were extracted using Nuclear and Cytoplasmic Protein Extraction Kit, and Western blotting was conducted by indicated antibodies. HADC1 was served as a nucleus marker and HSP70 was served as a cytoplasm marker. **(E)** Cell morphology in the BT549 cell line. N-Cadh, N-Cadherin; E-Cadh, E-Cadherin. Tubulin or β-actin was set as an internal control.

In order to further clarify the underlying molecular mechanisms of CD-inhibited cell migration and invasion, we tested the levels of the factors involved in EMT as well as different related EMT-TFs by CD treatment. After this, Western blotting was performed using antibodies against the proteins of interest. We first performed Western blotting to monitor the levels of factors involved in EMT in BT549 and 4T1 cells. **Figures 3A, B** show that TWIST1, SLUG, SNAIL1, and ZEB1 in both BT549 and 4T1 cells were dramatically downregulated by CD treatment. Then, we checked the protein levels of EMTs in these cells and found that CD is capable of down- and up-regulating N-Cadherin and E-cadherin, respectively (**Figures 3A, B**). To further clarify whether the changes in these markers are dose-dependent, different amounts of CD (0, 5, 10, 20 μM) were treated in BT549 cells, and results showed that CD down-regulated TWIST1, SLUG, SNAIL1, ZEB1, and N-Cadherin and up-regulated E-cadherin in a dose-dependent manner (**Figure 3C**). Since the EMT-related TFs are mostly nuclear, we conducted Western blotting to monitor whether EMT-TFs were reduced in nuclear localizations. To do so, BT549 cells were treated without or with indicated CD; proteins from the cytoplasm, nucleus, or total were extracted, and Western blotting was conducted. The results show that all TWIST1, SLUG, SNAIL1, and ZEB1 localized in the nucleus and the nuclear levels were reduced by CD treatments (**Figure 3D**). In addition, we have also observed that CD treatment affected the morphology of both BT549 (**Figure 3E**) and 168FARN (**Supplementary Figure 3**) but not 4T1 cells (data not shown).

**Figure 4:**
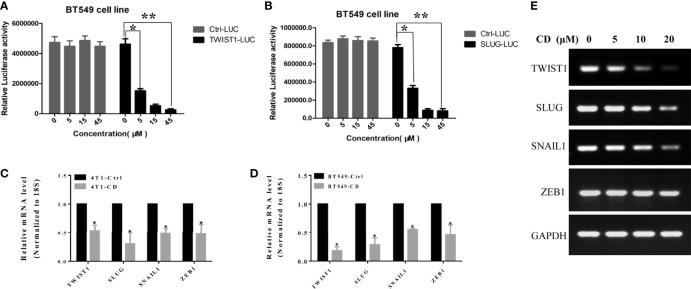
Effects of cordycepin on the TWIST1 or SLUG reporter activity. **(A)** Luciferase reporter assay showed that CD decreased the TWIST1 reporter activity in Hela cells, as the relative light unit (RLU) was decreased with the increase in CD dosage. **(B)** Luciferase reporter assay showed that CD reduces the SLUG reporter activity in Hela cells, as the Relative Light Units (RLU) were reduced with the increase of CD dosage. *P < 0.05, **P < 0.01. **(C, D)** The levels of mRNA for *TWIST1*, *SLUG*, *SNAIL1*, and *ZEB1* in 4T1 or BT549. 4T1 or BT549 cells were treated with 10 µM of CD for 24 h, total RNA was extracted, and quantitative RT-PCR analysis was performed. **(E)** The levels of mRNA for *TWIST1*, *SLUG*, *SNAIL1*, and *ZEB1* in BT549 were treated with the indicated concentration of CD. BT549 cells were treated with 0, 5, 10, and 20 µM of CD for 24 h, total RNA was extracted, and semiquantitative RT-PCR and quantitative RT-PCR analyses were performed.

To determine if these EMT-TFs were regulated by CD at the transcriptional level, luciferase reporter assay was performed by using *TWIST1* and *SLUG* genes. The promoter regions of these genes were inserted in front of the reporter gene, and the plasmid containing the reporter was transfected to the HeLa cells. The cells were then treated with or without CD for 48 h, and luciferase activities were measured. As shown in **Figure 4**, CD dose-dependently repressed the luciferase activities (relative light units, RLU) regulated by TWIST1 or SLUG promoter reporter (**Figures 4A, B**). Then, quantitative RT-PCR further confirmed that the levels of mRNA for *TWIST1*, *SLUG*, *SNAIL1*, and *ZEB1* were down-regulated by CD in both 4T1 and BT549 cells (**Figures 4C, D**). These regulations by CD were also shown in a dose-dependent manner (**Figure 4E**). These results strongly suggest that all of the TWIST1, SLUG SNAIL1, and ZEB1 were directly regulated by CD.

### Effects of Cordycepin on 4T1 Cell-Derived Allograft Tumors in Mice

**Figure 5:**
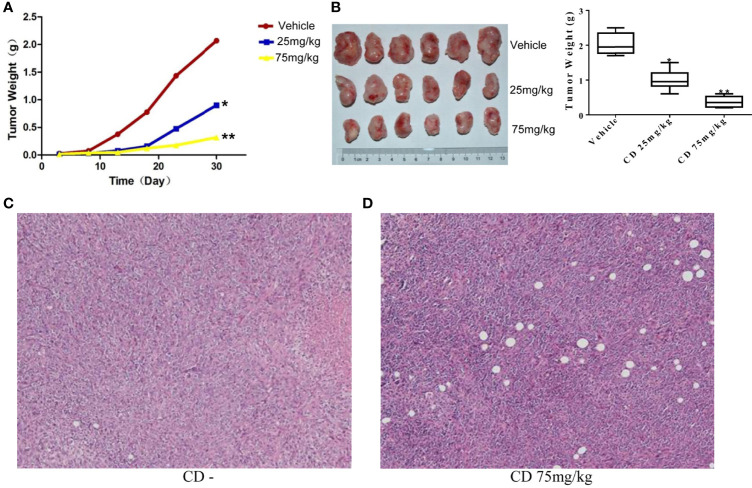
Cordycepin treatment inhibits tumor growth in breast cancer model of mouse. **(A, B)** CD treatment inhibited the tumor growth in mice. Quantitative data are shown in the right panel of **(B)**. **(C)** H&E staining image results of *in situ* tumor tissues without CD treatments. **(D)** H&E staining image results of *in situ* tumor tissues with CD treatments. *P < 0.05, **P < 0.01.

To further examine whether CD can repress the cell growth, migration, and invasion of TNBC *in vivo*, we decided to conduct a series of experiments with the congenic mouse tumor allograft model. First, to establish the animal breast cancer model, mouse breast cancer cells (4T1) were injected into the mammary fat pads of 18 female BALB/c mice. Three days later, the mice were randomly divided into three groups and treated with different levels of CD (25 or 75 mg/kg) as indicated in the legend of **Figure 5**. The sizes of the tumors were estimated every 5 days, and the results are presented in **Figure 5B**. The mice were sacrificed at the end of the 30-day treatment, the tumor tissues were dissected, the weight of the tumor tissues was measured, and the averages of the tumor weight are plotted as shown in **Figure 5A**. As shown in **Figures 5A, B**, compared to the control, the sizes and weights of the tumors were significantly decreased by CD treatment in a dose-dependent fashion. Moreover, CD-treated tissues of the *in situ* tumors showed nucleus pyknosis and perinucleus membranous vacuoles when compared without CD treatment control (**Figures 5C, D**).

**Figure 6:**
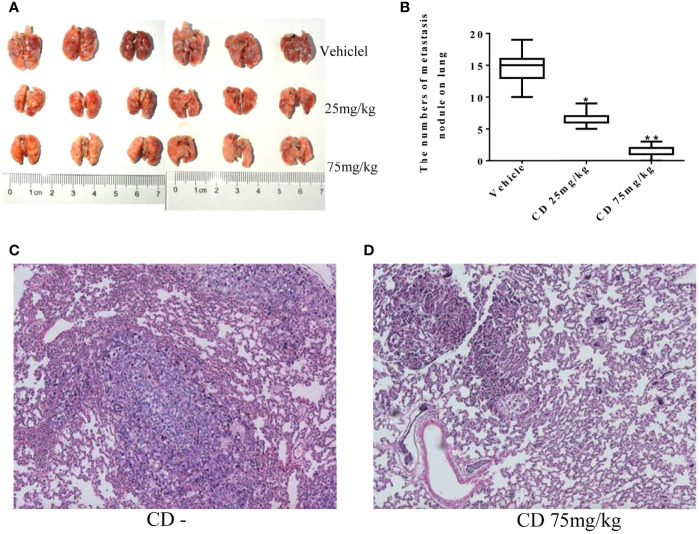
Cordycepin treatment inhibits cancer metastasis to lung. **(A)** The lung metastasis with different CD treatments. **(B)** Quantitative data from **(A)**. **(C)** H&E staining image results of lung tissues without CD treatments. **(D)** H&E staining image results of lung tissues with CD treatments. *P < 0.05, **P < 0.01.

Meanwhile, the CD effects on tumor cell migration were estimated by the number of colonies formed in the lungs. **Figure 6A** shows numerous colonies in the lungs of the non-treatment group, while only a few colonies were found in the CD-treated groups. The overall sizes of the colonies in the control group were clearly bigger than those in the CD-treated mice. Moreover, **Figure 6B** shows that the average number of colonies per mouse, which represent cancer cell migration/invasion, decreased in a dose-dependent fashion when the mice were treated with CD. **Figure 6D** shows CD-treated tissues of the lungs with a small metastatic size (arrows), while **Figure 6C** without CD treatment shows tissues of the lungs with large metastatic size (arrows). All of these *in vivo* data unambiguously demonstrated that CD can inhibit the growth, migration, and invasion of TNBC models.

### Additive/Synergistic Effect of Cordycepin and Thymoquinone on EMT-TFs

**Figure 7:**
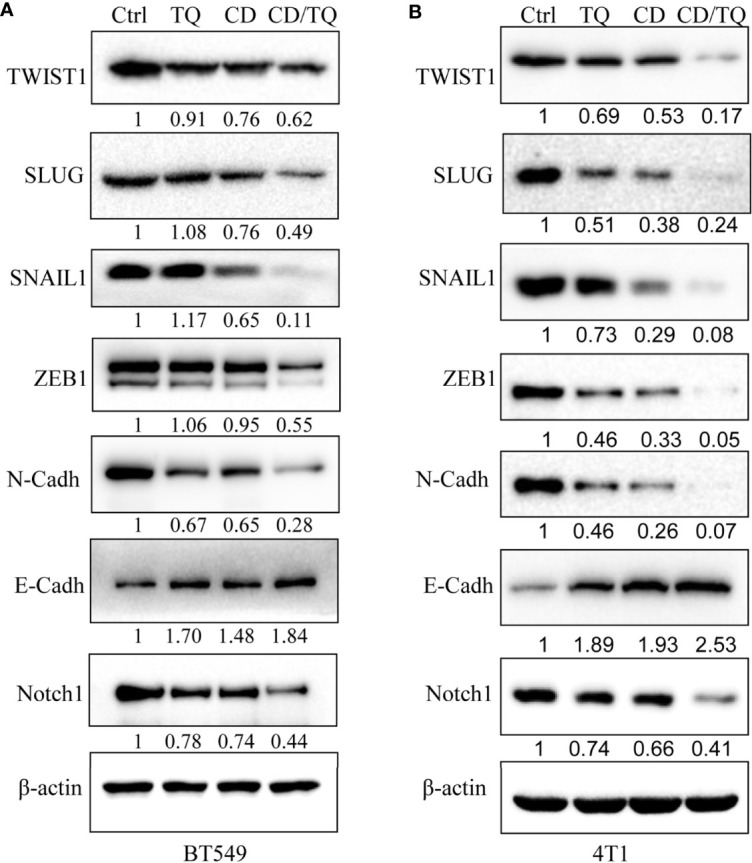
Synergistic effects of cordycepin and thymoquinone on the expression of EMT-TFs **(A)** and Notch **(B)**. CD (5 μM), TQ (5 μM), or both treatments for 24 h for Western blot by indicated antibodies. β-Actin was set as an internal control. Results are repeated twice. N-Cadh, N-Cadherin; E-Cadh, E-Cadherin.

Results from different labs including ours demonstrated that thymoquinone (TQ), another anticancer natural product, is capable of inhibiting breast cancer cell growth, migration, and invasion by targeting EMT-TFs. Given the similarities between the effects of TQ and CD, we decided to explore the potential additive/synergistic effects of these natural compounds on breast cancer progression and metastasis. To do so, we choose to treat the TNBC cell line BT549 with either 5 µM of CD or 5 µM of TQ alone or in combination of both. After 24 h of treatment, the levels of proteins involved in EMT were estimated by Western blotting with different antibodies. As expected, either CD or TQ alone is able to downregulate TWIST1, SLUG, SNAIL1, ZEB1, E-cadherin, and N-cadherin, while the combination of CD and TQ downregulated these proteins more potentially in BT549 TNBC cells (**Figure 7A**). Moreover, Notch1 is an important oncogenic factor and TQ could inhibit the expression. We have also tested the levels of Notch1 in BT549 cells; the results showed that either CD or TQ alone is able to downregulate, while the combination of CD and TQ downregulated Notch1 more strongly (**Figure 7A**). These data conclude that the combination of CD and TQ can repress breast cancer cell EMTs or other oncogenic factors additively or synergistically, suggesting that CD and TQ combination could repress breast cancer metastasis more efficiently than either molecule alone. In addition, we have also tested the levels of these proteins in mouse breast cancer line 4T1. As shown in **Figure 7B**, similar with BT549, either CD or TQ alone or the combination of both reagents was able to regulate these EMT and EMT-TFs. On the other hand, the combination of both was able to downregulate Notch1. These results are consistent with our previously reported findings.

## Discussion

Although a few primary cancers can lead to cancer-related death, the overwhelming majority of cancer-induced mortalities are due to the metastasis of the primary tumors. Despite great progress in cancer treatment in the last few decades, most metastatic cancers are still not curable. More importantly, there are extremely few options in treating TNBC mainly due to the limited understanding of the underlying mechanisms for metastasis of them. In order to conduct a systemic investigation of the possibility of developing CD as a potential therapeutic agent for TNBC, we first conducted *in vitro* experiments by treating two TNBC cell lines with different concentrations of CD and estimated its effects on cell growth, migration, and invasion. Consistent with the previous findings (19) about the anticancer and anti-metastatic activities of CD (2, 3, 6, 7, 20), we found that CD can not only inhibit growth but also repress both migration and invasion. We then took advantage of the 4T1 mouse allographic tumor model to substantiate the inhibitory effect of CD on breast cancer growth and metastasis *in vivo*. These results from both *in vitro* and *in vivo* experiments unambiguously demonstrated that CD is indeed capable of inhibiting different developing stages of TNBC including tumor cell growth, migration, and invasion.

The 4T1 cells were derived from a metastatic subpopulation of the spontaneously arisen BALB/c mouse mammary tumor. These cells can be injected into the mammary fat pads of the mice to establish an animal model for different experiments. In addition, when the 4T1 cells are allografted to the mammary glands of mice, local tumors are rapidly formed in the recipient animal and extensive lung metastasis is developed soon after. Therefore, this particular animal model was chosen, which enabled us to test the inhibitory effects of CD on different stages of TNBC *in vivo*. More importantly, instead of injecting cancer cells into immune-compromised mice, this TNBC model is established in mice with their intact immune system which plays important roles in cancer metastasis. Therefore, the results derived from experiments using this animal model would be more applicable for treatment of human patients.

It is well established that EMT plays indispensable roles in cancer metastasis. In order to understand the mechanisms of CD inhibition of cancer cell metastasis, we estimated the levels of the well-studied protein factors involved in EMT, such as E- and N-cadherins because the high expression in N-Cadherin and low expression in E-Cadherin are considered as hallmarks of EMT (11, 13, 21). We have also examined the levels of the EMT-TFs such as TWIST1, SNAIL, SLUG, ZEB1, and ZEB2 because targeting these factors has been suggested as potential cancer therapeutics (11, 13).

Our systemic evaluation found that the expression of these factors varies greatly among the cells examined. We found that TWIST1, SLUG, SNAIL1, and ZEB1 were readily detectable in both BT549 and 4T1 cells, and both of them were significantly downregulated by CD and the results from reported gene assays suggest that the CD-mediated downregulation of these TFs is at transcriptional levels. These findings are consistent with the results when hepatocellular carcinoma cells were treated with CD (22). Based on the report gene assays for TWIST1 and SLUG promoter and RNA levels of these EMT-TFs, we propose that CD could mechanically suppress their expression transcriptionally.

In previous studies, we have reported that another natural product TQ possesses anticancer and anti-metastatic activities partially by inhibiting EMT. In addition, TQ is also capable of down- and up-regulating N-cadherin and E-cadherin, respectively, *via* inhibiting TWIST1 and ZEB1 at the transcriptional level (15, 23). In this study, we demonstrated the additive/synergistic effect of CD and TQ on downregulation of TWIST1, SLUG, SNAIL1, ZEB1, Notch1, and N-cadherin in human TNBC cell line BT549 and mouse TNBC cell line 4T1. Altogether, these findings suggest that CD by itself and in combination with TQ possesses great potential to be developed as therapeutic drugs for TNBC treatment.

## Data Availability Statement

The original contributions presented in the study are included in the article/**Supplementary Material**. Further inquiries can be directed to the corresponding authors.

## Ethics Statement

The animal experiments followed the international, national, and institutional guidelines for the care and use. The study has been approved by the Ethical Committee of Southwest Medical University.

## Author Contributions

CW, MK, JC, JD, and MT conducted experiments. CW and MK prepared the figures. JC analyzed the data. CW and JD performed the CCK8 and Western blot assays. CW and JC performed the cell invasion and migration assay. CW and MK performed the mouse model assay. CW, MK, and MT wrote the manuscript. CW and JF revised the manuscript. JF and EL planned and supervised the project. All authors reviewed the manuscript. All authors contributed to the article and approved the submitted version.

## Funding

This work was supported in part by the National Natural Science Foundation of China (81672887, 82073263, 81172049), the Research Foundation of the Science and Technology Department of Sichuan Province, Science and Technology Innovation Team of Colleges and Universities of Sichuan Province (13TD0032), the Talent Startup Foundation of Southwest Medical University (00040150), and the Postdoctoral Startup Foundation of Southwest Medical University (00040182).

## Conflict of Interest

The authors declare that the research was conducted in the absence of any commercial or financial relationships that could be construed as a potential conflict of interest.

## Publisher’s Note

All claims expressed in this article are solely those of the authors and do not necessarily represent those of their affiliated organizations, or those of the publisher, the editors and the reviewers. Any product that may be evaluated in this article, or claim that may be made by its manufacturer, is not guaranteed or endorsed by the publisher.
