# Supplementary figures and images for: Cordycepin Inhibits Triple-Negative Breast Cancer Cell Migration and Invasion by Regulating EMT-TFs SLUG, TWIST1, SNAIL1, and ZEB1
Source: Front Oncol. 2022 Jun 14;12:898583. doi: 10.3389/fonc.2022.898583 (PMC9237498; doi:10.3389/fonc.2022.898583)

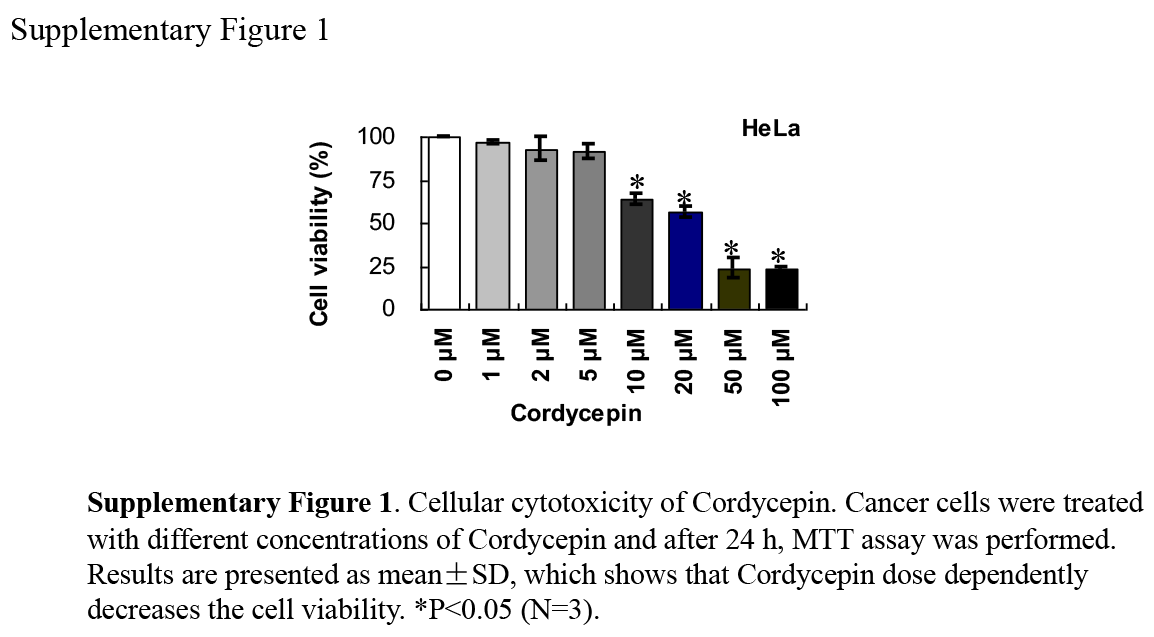

Supplement: Supplementary file 1 [file Image_1.tif]

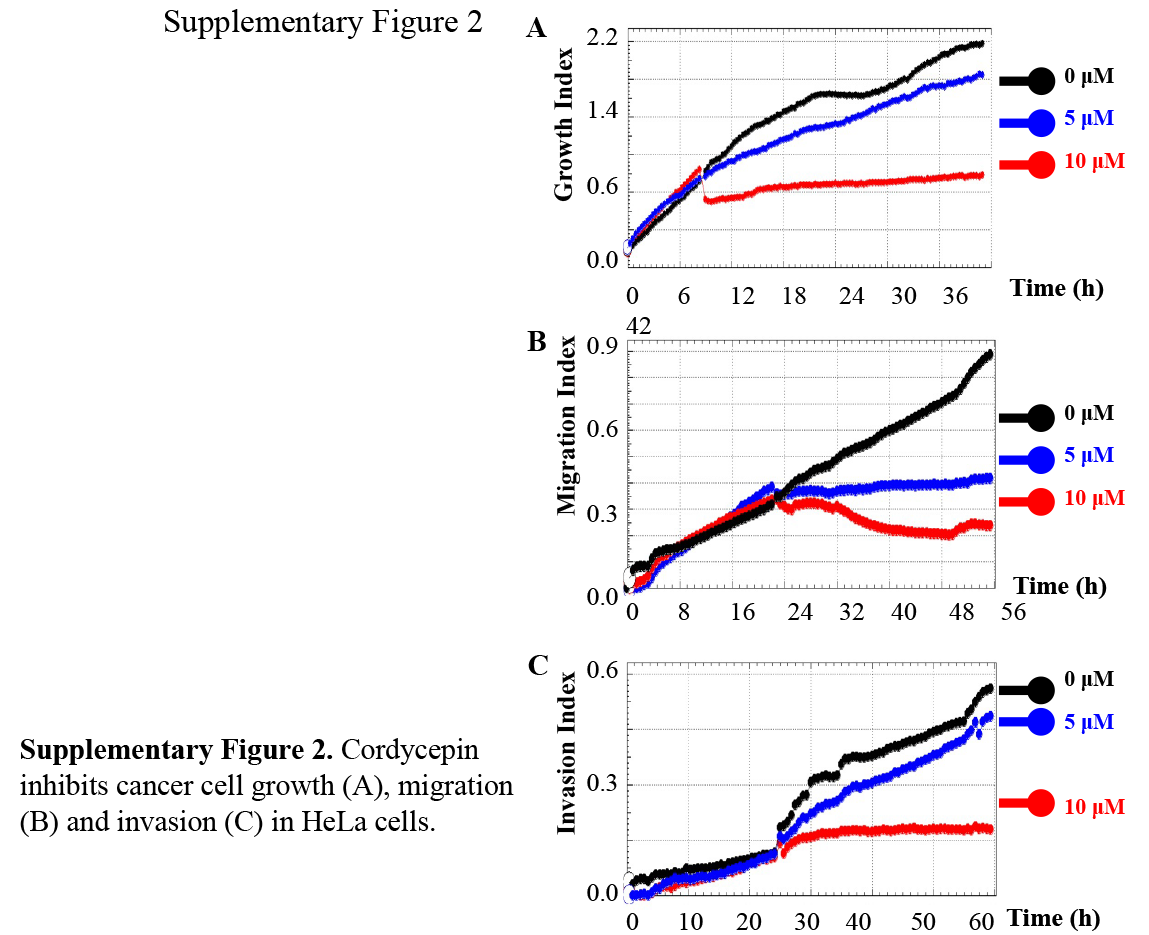

Supplement: Supplementary file 2 [file Image_2.tif]

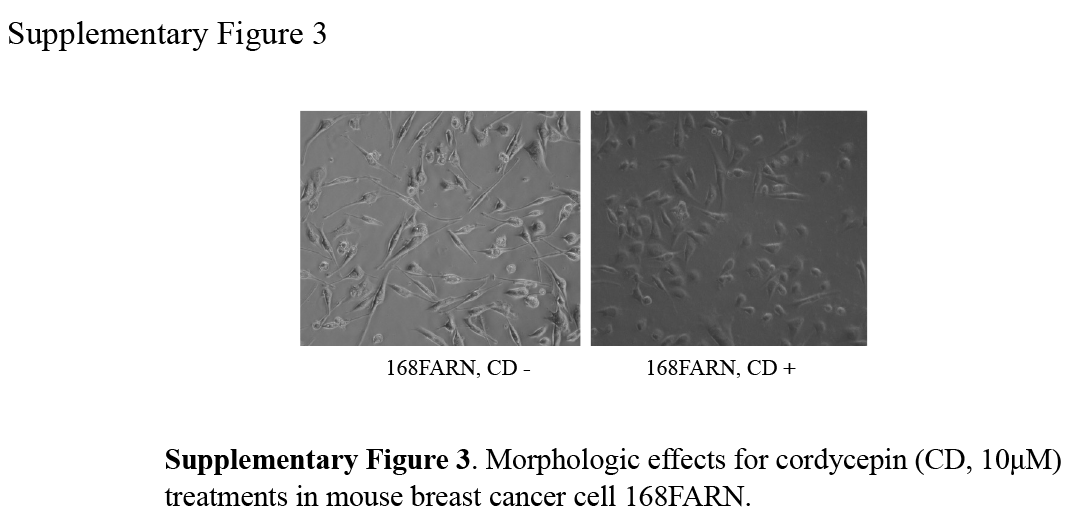

Supplement: Supplementary file 3 [file Image_3.tif]
